# The Effect of Germination and Fermentation on the Physicochemical, Nutritional, and Functional Quality Attributes of Samh Seeds

**DOI:** 10.3390/foods12224133

**Published:** 2023-11-15

**Authors:** Belal M. Mohammed, Isam A. Mohamed Ahmed, Ghedeir M. Alshammari, Akram A. Qasem, Abu ElGasim A. Yagoub, Mohammed Asif Ahmed, Abdullah A. A. Abdo, Mohammed Abdo Yahya

**Affiliations:** 1 Department of Food Science and Nutrition, College of Food and Agricultural Sciences, King Saud University, Riyadh 11451, Saudi Arabia; azzomor.b@gmail.com (B.M.M.); aghedeir@ksu.edu.sa (G.M.A.); aqasem@ksu.edu.sa (A.A.Q.); amohammed4@ksu.edu.sa (A.E.A.Y.); masifa@ksu.edu.sa (M.A.A.); mabdo@ksu.edu.sa (M.A.Y.); 2 Beijing Advanced Innovation Center for Food Nutrition and Human Health, Beijing Technology and Business University, Beijing 10048, China; drabdoabdullah2021@gmail.com

**Keywords:** bioactive properties, fermentation, functional properties, germination, samh seeds

## Abstract

This study investigated the effects of fermentation and germination on the physicochemical, nutritional, functional, and bioactive quality attributes of samh seeds. Regardless of the processing treatment, samh seeds were found to be a rich source of phenolic compounds, namely gallic acid (79.6–96.36 mg/100 g DW), catechol (56.34–77.34 mg/100 g DW), and catechin (49.15–84.93 mg/100 g DW), and they possessed high DPPH antiradical activity (65.27–78.39%). They also contained high protein content (19.29–20.41%), essential amino acids content (39.07–44.16% of total amino acids), and unsaturated fatty acid content (81.95–83.46% of total fatty acids) and a low glycemic index (39.61–41.43). Fermentation and germination increased L*, b*, foaming capacity, oil absorption capacity (OAC), water absorption capacity (WAC), swelling power, microbial counts, antioxidant activity, total flavonoid content (TFC), total phenolic content (TPC), in vitro protein digestibility, protein efficiency ratio, and total essential amino acids and reduced water solubility, emulsion stability, tannin, and phytate contents compared to raw samh seeds (*p* < 0.05). The highest levels of pH, ash, carbohydrate, fiber, and glycemic index were observed in raw samh seeds, and both germination and fermentation processes reduced these attributes to various degrees (*p* < 0.05). Germination increased the redness (a*), moisture content, essential and non-essential amino acids, potassium, zinc, phosphorous, stearic acid, and oleic and unsaturated fatty acids and reduced total solids, fat content, iron, zinc, calcium, magnesium, sodium, palmitic acid, and total saturated fatty acids of the samh seeds compared to the raw ones. Fermentation increased the total solid, acidity, fat, protein, calcium, magnesium, sodium, phosphorous, iron, zinc, palmitic acid, and total saturated fatty acids and reduced the a* value, moisture, non-essential amino acids, and total unsaturated fatty acids of the samh seeds compared to the raw ones. In conclusion, samh seeds are a rich source of nutrients that could generally be enhanced by germination and fermentation processes. The reported information facilitates strategies towards the application of these underutilized seeds in foods.

## 1. Introduction

Recent decades have witnessed increased interest in the production and utilization of gluten-free food products from underutilized plant sources to overcome the health concerns associated with gluten-containing foods and to feed the growing world population [1]. In addition, global warming and shortage of water supply have also directed the research toward utilizing tolerant plants as potential sources of food, feed, cosmetic, and pharmaceutical products. In this regard, the samh (*Mesembryanthemum forsskalei* Hochst) plant is considered a suitable candidate that could be exploited in food and pharmaceutical applications. Samh is a halophyte plant that grows naturally in the semi-arid zones of Northern Africa and the Middle East and in many countries around the world [2,3]. Samh is extremely tolerant to harsh desert environments, such as elevated temperatures, the salinity of soil and water, and limited water resources [3,4]. This has subsequently led to demands for its cultivation and utilization to increase in recent years. People in the Al-Jouf area of northern Saudi Arabia [1] commonly use the seeds of Forskal Fig-marigold and locally call it “Samh”. Bedouins harvest the seeds by soaking the dried fruits in water to allow for the opening of the fruit valves and the release of the seeds, which precipitate to the bottom of the container. Samh seeds are used in several food, feed, and pharmaceutical applications [5]. Traditionally, Bedouins in Saudi Arabia use samh seeds and flours for the preparation of numerous local dishes namely Al-Bekailah (dates with samh flour and butter), Al-Batholelaah (ground roasted samh), Al-Baseesah (samh flour and butter or oil), Aseedah (samh flour cooked in water with added milk or butter), Al-Muthgalah (cooked samh and wheat flour with added butter), and samh bread [4]. Additionally, several types of breads, sweets, and cookies are prepared from samh seed flour [4,6]. Nutritionally, samh seeds are a high-energy (>3000 kcal/serving) food, are considered a high-quality protein source (>21%), like legumes and pulses, have considerable fat content (majorly, unsaturated fatty acids) and substantial amounts of minerals, amino acids, and fiber and are thus considered as part of a balanced diet [1,4,5,7,8]. In addition, they are a rich source of flavonoids, phenolics, and phytosterols that exhibit antioxidant, antibacterial, antifungal, and phytotoxic activities [9,10,11]. Studies utilizing animal models have indicated that samh seeds possess several pharmaceutical and health-promoting properties [9,12,13,14,15]. In this regard, a rats’ feeding trial that used samh seeds at a 15% replacement level reported lower cholesterol, glucose, triglyceride, creatinine, and LDL-C concentrations in the rats’ blood in the samh-treated group [12]. In addition, feeding diabetic and obese rats with 5% samh seeds maintained low levels of blood glucose, triglycerides, and cholesterol and regulated the rats’ body weight, which suggested that the seeds are particularly useful for diabetic patients [14]. Moreover, samh seeds improved the lipid profile and antioxidant enzymes in diabetic rats [13]. Furthermore, samh seeds were also found to prevent liver injury through their downregulation of oxidative stress [15]. The above-mentioned studies indicate that samh seeds exhibit antioxidant, antihyperlipidemic, anti-diabetic, and hepatoprotective effects, and thus, these seeds are a potential candidate for food and medicinal applications.

Prior to consumption and use, samh seeds undergo several processing treatments to improve their sensory attributes and nutritional quality. Roasting and cooking are the main thermal processes of samh seeds [5,16]. Roasted seeds are milled to form fine flour and then applied as a supplement or substitute to wheat flour in bakery products such as bread and cookies to enhance their nutritional and sensory attributes [4,6]. In addition, samh seeds have been used to replace corn in poultry feed due to their high nutritional quality [17]. However, like other grains, complete utilization and capture of the benefits of samh seeds are hindered by the existence of antinutritional factors such as phytates, tannins, and trypsin inhibitors [16]. Antinutritional factors are known to affect the bioavailability of essential minerals such as iron, zinc, potassium, and phosphorus [18]. Therefore, processing methods are usually used to diminish the antinutritional factors and improve the nutritional, health, and sensory attributes of plant seeds [16]. Of these processes, roasting, boiling, baking, and cooking have been commonly used to reduce the antinutritional factors, enhance the chemical composition and in vitro and in vivo protein digestion, improve biological value, and consequently enhance the nutritional and health properties of samh seeds [16]. Roasting treatment was found to improve the antioxidant activity and phenolic acids, flavonoid, linoleic acid, potassium, and calcium contents of samh seeds [5]. Non-thermal processing, such as germination and fermentation, are powerful methods that could be used to improve the nutritional, sensorial, and health quality of foods. They have the ability to create unique food products with special quality and functionality and reduce harmful ingredients in plant seeds. However, these methods have not been applied to samh seeds. Therefore, the main goal of this study is to investigate the influence of fermentation and germination on the fatty acids, amino acids, minerals, and physicochemical, functional, and bioactive quality attributes of samh seeds.

## 2. Materials and Methods

### 2.1. Materials

The seeds of the samh plant (*Mesembryanthemum forsskalei* Hochst) were harvested during the 2022 growing season from the Al-Jouf district, northern Saudi Arabia. The seeds were physically cleaned to remove dirt and impurities and stored in firmly closed containers at 4 °C until being used for germination, fermentation, and chemical analyses. For the preparation of raw flour, part of the samh seeds was ground into fine flour by a grinder (Moulinex coffee, AR11, Beijing, China), and the flour was stored refrigerated at 4 °C in polyethylene bags for further use. The chemical reagents and standards used were of analytical grade and were acquired from Sigma-Aldrich (St. Louis, MO, USA).

### 2.2. Germination Process

**Figure 1:**
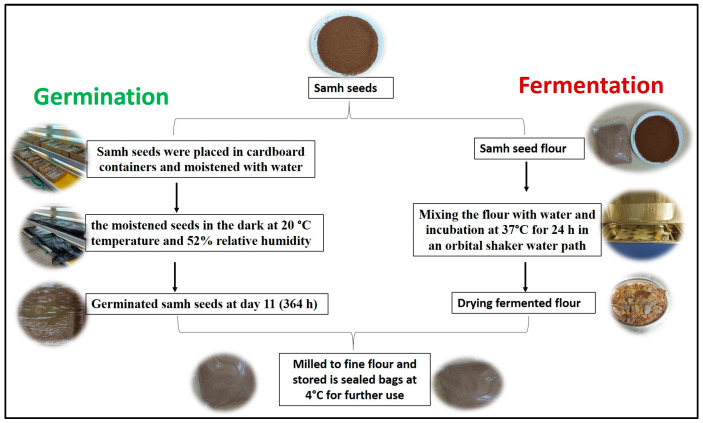
Fermentation and germination processes of samh seeds.

Germination of the samh seeds was carried out as stated by Mahmoud et al. [19] with some modification. Germination was conducted in pre-disinfected cardboard containers at 20 °C temperature and 52% relative humidity in a dark atmosphere for eleven days (Figure 1). Briefly, the seeds were spread on wet large brown kraft mailing cartoon boxes (18 × 12 × 3 Inches), covered with a kitchen towel, and moistened with water every 24 h until a full germination process was achieved (264 h). Then, the sprouts were subjected to a drying process at 40 °C for 48 h when a constant weight was achieved. The dry sprouts were ground into fine flour by a grinder (Moulinex coffee, AR11, Beijing, China), and the flour samples were stored refrigerated at 4 °C in polyethylene bags for further use.

### 2.3. Fermentation Process

The samh seeds were fermented for 24 h following the process of Carciochi et al. [20] with some modification. Prior to fermentation, the samh seeds were milled by a grinder (Moulinex coffee, AR11, Beijing, China) into fine powder to pass through a 0.5 mm sieve. For fermentation, samh flour was added to sterile ddH_2_O at the ratio of 1:2 (*w*/*v*) in covered Erlenmeyer flasks and mixed. After that, the blend was subjected to 24 h incubation at 37 °C in an orbital shaking incubator (HS-B 20 D, IKA Labortechnik, Staufen, Germany) to allow indigenous microorganisms (natural fermentation) to grow and ferment the mixture (Figure 1). During the fermentation process, an aliquot of 50 mL was taken from the fermentation mixture at 8 h intervals to measure pH (AOAC method number 981.12) and total acidity (AOAC method number 942.15) [21]. Then, the fermented samples were dried at 40 °C to a consistent weight, followed by milling into fine flour, and the flour samples were stored in sealable polyethylene bags at 4 °C for further use.

### 2.4. Surface Color Examination

A NIX Pro 2 Color Sensor was used to test color values (Hamilton, ON, Canada). Before measurement, the device was calibrated using a white surface calibration plate. The CIE color parameters (lightness (L*), redness (a*), and yellowness (b*)) were recorded for each sample in triplicate.

### 2.5. Functional Properties Examination

#### 2.5.1. Water and Oil Absorption Capacity

According to the procedures outlined by Sosulski et al. [22], 1 g of each type of samh seed flour (raw, fermented, and germinated) was combined with 10 mL of ddH_2_O or refined sunflower oil, thoroughly mixed, and left for 30 min at room temperature. In weighed centrifuge tubes, the resultant blends were subjected to 10-min centrifugation at 2000× *g* followed by decanting of the supernatants. The difference between the sample’s initial weight and its weight after the water or oil addition had been emptied was used to determine OAC and WAC. The results are presented as grams of bound water or oil per gram of samh seed flour.

#### 2.5.2. Emulsification Properties

The emulsifying abilities of raw, fermented, and germinated samh seed flour were assessed using Elkhalifa and Bernhardt’s [23] method with slight modification. A high-speed lab blender was used to combine 2 g of samh seed flour with 20 mL of water. During mixing, refined sunflower oil was progressively added (0.4 mL/s) until phase separation took place. The emulsification activity (EA) was determined as the ratio of the emulsified layer to the overall volume. The emulsion underwent a second centrifugation step after subjecting it to heating treatment at 80 °C for 30 min followed by cooling to 15 °C. The percentage of the entire volume that remained emulsified after heating was considered as the emulsion stability (ES).

#### 2.5.3. Foaming Properties

The foaming capacity (FC) and foam stability (FS) of the raw, fermented, and germinated samh seeds were determined using the technique of Figueroa-Gonzalez et al. [24]. The samh flour samples and water (0.7 g in 100 mL, *w*/*v*) were blended in a high-speed lab mixer for 5 min, after which the mixture was decanted into a measuring cylinder (250 mL) and the foam volume was measured and used for calculation of the percentage of foaming capacity. The percentage of foam volume in the measuring cylinder remaining after 60 min of incubation was considered as the foaming stability.

#### 2.5.4. Bulk Density, Swelling Power and Solubility

The bulk density of samh seed samples (raw, fermented, and germinated) was assessed using a documented procedure [25] with slight alterations. A graduated cylinder (10 mL) containing 1.5 g of sample was weighed and the cylinder was moderately tapped until there was no further reduction in the sample level, and the bulk density (g/cm^3^) was determined by dividing the sample weight by the sample measured volume. The swelling power and water solubility of the samh seed samples (raw, fermented, and germinated) were assessed using published methods [26]. Briefly, a 0.2 g sample was combined with 10 mL of ddH_2_O in pre-weighed centrifugal tubes and the blend was subjected to 30 min of heating at 90 °C. Then, the mixture was centrifuged for 15 min at 3000× *g*. After decanting the supernatant, the increase in the sample’s weight was determined as the swelling power. The soluble matter-containing decanted supernatant was dried in dishes that had already been precisely weighted, and the residual weight was then expressed as the water solubility.

### 2.6. Acidity, pH, Total Solids, and Proximate Composition Examination

The standard AOAC official methods numbers 942.15, 981.12, 925.09, 940.09, 942.05, 992.15, 991.43, and 960.39 [21] were used for the determination of the acidity, pH, total solids, fat, protein, ash, moisture, and fiber contents of the raw, fermented, and germinated samh seeds. The total carbohydrates were measured by deducting the sum of all proximate attributes (% moisture + % crude fat + % crude protein + % ash) from 100%.

### 2.7. Microbial Load Examination

The total viable count (TVC) and mold and yeast counts were assessed using Oxoid nutrient agar and potato dextrose agar, respectively [27]. Optimally diluted samples of raw, fermented, and germinated samh seed flour in sterile peptone water were plated on sterile nutrient agar media (TVC) or potato dextrose agar media (yeast and mold) and incubated for 24 h at 37 °C (TVC) or for 2–7 days at 25 °C (yeast and mold counts). The plates were investigated for microbial growth and the results were specified as log CFU/g.

### 2.8. Glycemic Index Examination

The glycemic index of raw, fermented, and germinated samh seeds was estimated following a previously reported method [28] with some alterations. Concisely, a 0.1 g sample was added to 2 mL of 50 mM HCl solution containing pepsin (117 mg/mL) and the mixture was subjected to 30-min incubation at 37 °C under continuous shaking. Then, 4 mL of 500 mM NaCH_3_COO buffer (pH 5.2), 1 mL of pancreatin (243 mg/mL), and 56 μL amyloglucosidase (260 U/mL) were added to the mixture followed by 180-min incubation at 37 °C under continuous shaking. At 20 min intervals, 100 μL aliquots were taken and added to 1 mL ethanol followed by 10-min centrifugation at 8000× *g*. Then, a glucose determination kit, GOD-PAP (Fortress Diagnostics Ltd., Antrim, UK), was used for the determination of glucose content spectrophotometrically at 500 nm (Shimadzu UV-1800, Kyoto, Japan). A hydrolysis curve was generated using the absorbance against the time and used for the calculation of hydrolysis and glycemic indexes.

### 2.9. Bioactive Properties Examination

#### 2.9.1. Preparation of Aqueous Methanolic Extract

Prior to the analysis of the bioactive properties of raw, fermented, and germinated samh seeds, extracts were prepared as reported previously [5]. Concisely, a 1 g samh seed sample was added to 10 mL methanol (80%), and extraction was carried out using an ultrasonic water bath for 30 min, followed by 10-min centrifugation at 8000× *g*. The extraction process was repeated three times, and the obtained supernatants were combined, concentrated in a rotary evaporator at 37 °C, filtered with filter membranes (0.45 μm), and then retained for further use.

#### 2.9.2. Estimation of the Bioactive Compounds of Samh Seed Extracts

The total phenolic content (TPC) of the samh seed extracts was assessed by using the Folin–Ciocalteu (FC) reagent colorimetric method [29]. After 5 min of incubation at room temperature, the reaction mixture of equal volume of sample extract and FC reagent (1:1, *v*/*v*) was mixed with 10 mL of Na_2_CO_3_ (700 mM) and ddH_2_O in the total volume of 25 mL. After an additional 1 h of incubation at room temperature, the absorbance was read at 750 nm (Shimadzu UV-VIS, Japan Spectrophotometer, Kyoto, Japan) and the results were reported as mg gallic acid equivalents per 100 g (mg GAE/100 g) using the standard curve generated with gallic acid (0–200 mg/mL).

The total flavonoid content (TFC) of raw, fermented, and germinated samh seed extracts was assessed using Dewanto et al.’s [30] method. After 5-min incubation at room temperature of the mixture of 1 mL extract and 0.3 mL of 720 mM NaNO_2_, 0.6 mL of AlCl_3_ (750 mM) was added and the reaction mixture was subjected to further incubation for 6 min at room temperature followed by the addition of 2 mL of NaOH (1 M) and ddH_2_O to a final amount of 5 mL. The absorbance of the pink color solution was recorded at 510 nm, and the results are presented in mg catechin equivalents per 100 g (mg CE/100 g) samh seeds.

The individaul phenolic compounds of the raw, fermented and germinated samh seed extracts were separated, identified, and quantified using the HPLC method as described previously [5].

#### 2.9.3. Antioxidant Activity

The antioxidant activity of the raw, fermented, and germinated samh seeds extracts was measured by using 1,1-diphenyl-2-picrylhydrazyl (DPPH) radical scavenging [5], ferric reducing antioxidant power (FRAP) [31], and 2,2-Azino-bis-3-ethylbenzothiazoline-6-sulfonic acid (ABTS) [32]. For DPPH antiradical activity, 0.5 mL extract reacted with 1 mL of DPPH solution for 30 min at room temperature prior to recording the absorbance at 517 nm against the blank, and the difference in the absorbance between the blank and the extract was used for calculation of the DPPH inhibition percentage. For the FRAP assay, 0.1 mL of sample extract was added to 1.5 mL of FRAP reagent (containing 0.3 M NaCH_3_COO buffer (pH 3.6), 0.01 M 2,4,6-Tri (2-pyridyl)-s-triazine (TPTZ) solution, and 0.02 M FeCl3.6H_2_O at a ratio of 10:1:1 (*v*:*v*)), and the reaction was allowed to proceed at 37 °C for 30 min. After that, the absorbance was recorded at 593 nm, and the results were specified as the amount of substance produced during the reduction of Fe^3+^-TPTZ to Fe^2+^-TPTZ. For the ABTS assay, 10 mL of 7 mM ABTS was combined with 10 mL of K_2_S_2_O_8_ (2.4 mM) in the dark for 2 days at room temperature to make the ABTS reagent. Immediately before use, 5 mL of the ABTS reagent was diluted with 100 mL ethanol to attain an absorbance of 0.70 at 734 nm. Then, 50 μL of sample extract was added to 2 mL of the ABTS solution, which was combined with 1.95 mL of ethanol to make a final volume of 4 mL. After 6-min incubation at room temperature, the absorbance was recorded against a blank at 734 nm, and the difference in the absorbance between the sample extract and the blank was used for calculation of the ABTS inhibition percentage.

### 2.10. Antinutritional Factors Measurement

The antinutritional factors of the raw, fermented, and germinated samh seeds were assessed by spectrophotometric measurement of phytate content [33] and tannin content [34]. For the phytate content assay, a 2 g sample was subjected to 5 h hydrolysis with 20% HCl (100 mL) followed by filtration with Whatman filter paper No. 2 and collection of the filtrate in a 25 mL conical flask. After that, 5 mL of an ammonium thiocyanate solution (0.3%) was added, and the mixture was then subjected to titration against FeCl_3_ solution until a brownish–yellow tint developed and remained for 5 min. Then, the results were recorded and used for the calculation of phytate based on the fact that an equal molar (1:1) ratio of Fe to phytate exists. The concentration of FeCl_3_ was 1.04% *w/v*, and the results were converted to mg phytic acid/g dry matter. For the tannin assay, 1 g of samh seeds (raw, fermented, or germinated) was mixed with 40 mL of 10% aqueous ethanol, and the mixture was heated in a water bath for one to two hours while being occasionally stirred. After filtering, the mixture was diluted with methanol to 50 mL. After that, 1 mL of the extract was mixed with 5 mL of Folin–Denis reagent, 10 mL of 35% saturated sodium carbonate, and 100 mL of distilled water, followed by 30-min incubation of the reaction mixture at room temperature. Then, the absorbance of the mixture was recorded at 760 nm, and results are reported as mg tannic acid equivalent (mg TAE/g sample).

### 2.11. Amino Acids and In Vitro Protein Digestibility Examination

The analysis of the amino acid profile of the raw, fermented, and germinated samh seeds was carried out as described by Mohapatra et al. [35]. The hydrolysis of the samples was carried out by mixing a 1 g sample with 5 mL of 6 M HCl in an airtight glass ampoule, and the mixture was heated at 100 °C for 24 h followed by filtration, dilution with ddH_2_O, drying using a rotary evaporator, and reconstitution with 0.1 N HCl (2.5 mL). After derivatization and membrane filtration (0.22 μm), a 20 μL sample was introduced to the HPLC system (Agilent 1260) furnished with an Eclipse Plus C18 column (4.6 mm × 250 mm × 5 μm) and DAD (338 nm) and fluorescence (260–340 nm excitation/305–450 nm emission) detectors. The mobile phase contained sodium phosphate buffer pH 7.8 (A) and acetonitrile: methanol: water 45:45:10 (B) was run at a 1.5 mL/min flow rate. A linear gradient mode of 98% (A) and 2% (B) from 0–33.40 min, 43% (A) and 57% (B) from 33.40–33.50 min, 0% (A) and 100% (B) from 33.50–39.40 min, and 2% (A) and 98% (B) from 39.40–40.0 min was used, and a constant temperature of 40 °C was maintained for the column. The equations described by Mohapatra et al. [35] were used for the calculation of the ratio of the essential amino acids to the total amino acids and the predicted protein efficiency ratio (P-PER).

The in vitro protein digestibility of the raw, germinated, and fermented samh seeds was assessed after 2 h hydrolysis at 37 °C using a reaction mixture composed of 0.4 g of samh sample and 1 mg of pepsin in 15 mL of 0.1 M HCl [36]. After termination of the reaction with 15 mL of 10% TCA, the mixture was subjected to 5-min centrifugation at 6300× *g* followed by filtration and quantification of the nitrogen content in the TCA soluble fraction by using the micro-Kjeldahl method [21]. Then, the percentage of protein digestibility was calculated by dividing the nitrogen content of the TCA filtrate by the nitrogen of the sample multiplied by 100.

### 2.12. Free Mineral Content Measurement

The free mineral content of the raw, fermented, and germinated samh seeds was analyzed using the method stated by Milani et al. [37]. Concisely, a 0.5 g sample was digested with 1 mL of concentrated H_2_SO_4_ at 220 °C for 30 min in a hot plate digestion system. After cooling to room temperature, 1 mL H_2_O_2_ was added, and digestion was continued at 400 °C until a clear solution was achieved (2 h). After digestion, Whatman #42 filter paper was used for filtration of the mixture, and the filtrate was diluted to a 50 mL final volume with ddH_2_O. Then, all micro- and macro minerals in the digested sample filtrates were analyzed by using an ICP-OES (Optima 4300 DV, Perkin Elmer, Waltham, MA, USA) except sodium and potassium, which were determined by a flame photometer (AE-FP 8501, Staffordshire, UK) and phosphorus which determined the content calorimetrically.

### 2.13. Fatty Acids Determination

Oil was extracted from the raw, fermented, and germinated samh seeds samples by using n-hexane in a Soxhlet oil extraction system for 8 h followed by methylesterification with methanolic boron trifluoride (BF3-MeOH) to form fatty acid methyl esters (FAMEs) as described previously [38]. The FAMEs of the samh seeds oil were separated using a DB-5 ms capillary column (30 m × 0.25 mm × 0.25 µm film thickness) connected to a gas chromatography–mass spectrometry (GC-MS) system (Agilent Technologies, Santa Clara, CA, USA) as described previously [5].

### 2.14. Statistical Analysis

Triplicate replicates were conducted for each treatment, the samples were analyzed in triplicate for each experiment, and the data were subjected to statistical analysis using SPSS software (version 20.0, SPSS, Chicago, IL, USA). Differences among the experimental groups were analyzed using ANOVA and Duncan’s multiple range test (DMRT), and significance was accepted at *p* < 0.05. All results are presented as the mean ± standard deviation.

## 3. Results and Discussion

### 3.1. The Effect of Fermentation and Germination on the Color and Functional Properties of Samh Seeds

The changes in the surface color and functional properties of samh seeds as influenced by the germination and fermentation process are shown in Table 1. Both treatments affected the color attributes in different manners (*p* < 0.05). In comparison to the control (raw samh seeds), germination increased the L*, a*, and b* values, whereas fermentation increased the L* and b* values and reduced the a* value (*p* < 0.05). The uppermost levels of L* and b* were seen in the fermented samh seeds, whereas the highest value for the a* value was noted in the germinated samh seeds. The increase in L* and b* of the germinated and fermented samh seeds is likely due to the enzymatic breakdown of intact colorant compounds during germination and fermentation processes. The surface color of the samh seed coat varies between yellow to deep red, and this might be an additional reason for the increased redness and yellowness of samh seeds during germination due to increased migration of the seed coat color to the endosperm where subsequent drying and milling processes concentrate these pigments in the seeds. The reduction in the redness of fermented samh seeds is probably due to the breakdown of the seed pigment during the fermentation process. Previous reports have shown varied changes in the color attributes of seeds and grains during germination and fermentation processes. For example, Tian et al. [39] reported an increase in redness and yellowness and a reduction in lightness during the germination of oat seeds and attributed these changes to the hydrolysis of protein and starch and the non-enzymatic Millard reaction. Sharma and Sharma [40] reported that germination and fermentation treatments increased the lightness and redness and reduced the yellowness of foxtail millet and ascribed this to the oxidation of pigmented compounds that led to brighter flour, and the passage of pigmented compounds from the seed coat to the endosperm may have led to augmented redness in the flour. The variations in color changes during fermentation and germination treatments could be accredited to the changes in the substrates and treatment conditions used in these processes.

Regarding the functional properties, both treatments significantly increased the oil absorption capacity (OAC), water absorption capacity (WAC), swelling power, and foaming capacity and decreased the water solubility and emulsion stability compared to the raw samh seeds (*p* < 0.05). Fermentation treatment greatly increased all of the assessed functional properties to the highest values, except for emulsion stability and water solubility, which were reduced in the fermented samh seeds compared to the raw ones (*p* < 0.05). The germination process considerably (*p* < 0.05) reduced the emulsion activity, foaming stability, and water solubility to the minimum values compared to that of the raw and fermented samh seeds. The alterations in the functional characteristics of the germinated and fermented samh seeds are probably due to the enzymatic and non-enzymatic structural modifications of macromolecules during the fermentation and germination processes. The rise in WAC might be due to the degradation of macromolecules such as polysaccharides and fiber and changes in protein content and quality, thereby increasing the hydrophilic sites and trapping more water within the flour matrix [41]. The upsurge in OAC in the germinated and fermented samh seeds is likely due to the unfolding of proteins and the decomposition of starch during germination and fermentation processes, which lead to increased hydrophobic amino acids and lipophilic compounds and thereby increase the OAC of the flour [42]. The higher WAC and OAC of the fermented and germinated samh seeds could improve flavor retention and mouth feel and indicate the potential applications of fermented and germinated samh seeds in the food industry. In agreement with our findings, increases in WAC and OAC were reported in fermented and germinated finger millet [42], germinated desi chickpea [43], germinated sorghum [23], and fermented African yam bean flour [44]. The upsurge in the foaming capacity of the fermented and germinated samh seeds might be due to changes in protein structure and quantity, and similar changes in foaming capacity were reported in germinated Bambara groundnut [45] and sorghum [46] and in fermented *Moringa oleifera* seeds [47]. The increase in swelling power during the fermentation and germination of samh seeds could be attributed to the energetic vibration among starch molecules, and therefore, the hydrogen bonds of starch could attract greater numbers of water molecules [46]. In agreement with our findings, it has been shown that germination and fermentation increased the swelling power of pearl millet [42] and foxtail millet [40]. The reduction in the water solubility, foaming stability, emulsification activity, and emulsion stability of the germinated samh seeds is likely due to the structural conformation, modifications in the protein molecules, and changes in fat and protein content [40]. Similarly, various reports have demonstrated that the fermentation and/or germination processes of grains and seeds reduce and/or increase the possessed water solubility, foaming stability, and emulsification activity and emulsion stability [40,45]. Overall, the germinated and fermented samh seeds possessed improved techno-functional characteristics and could be a potential candidate for food applications such as confectionaries and cookies.

### 3.2. The Effect of Fermentation and Germination on the Total Solids, pH, Acidity, Microbial Load, Proximate Composition, Fiber, and Glycemic Index of Samh Seeds

The results of the total solids, acidity, pH, total viable count (TVC), yeast and mold count, proximate composition, and estimated glycemic index of the samh seeds as influenced by the fermentation and germination processes are presented in Table 2. The germination and fermentation treatments affected these traits in different manners (*p* < 0.05). The fermentation process significantly increased the total solids, acidity, TVC, yeast and mold count, fat, and protein content to maximum values and reduced the pH, moisture, fiber, and estimated glycemic index of the samh seeds to the minimum values (*p* < 0.05). The highest values of pH, ash, carbohydrates, fiber, and glycemic index were observed in the raw samh seeds, and both process reduced these attributes to various degrees (*p* < 0.05). In comparison to the control, germination (*p* < 0.05) decreased the total solids and fat and augmented the moisture content of the samh seeds. The microbial count (TVC and yeast and mold count) was greatly increased by the fermentation and germination processes compared to the control. The reduction in pH and increase in acidity in the germinated and fermented samh seeds might be due to the increase in TVC and yeast and molds, and their increased metabolic activity could lead to the formation of organic acids. The increase in protein content in the germinated and fermented samh seeds is likely due to the synthesis of enzymes, the degradation of antinutritional factors, the synthesis of new proteins, and the digestion of insoluble storage proteins into simple soluble proteins [41,45]. In accordance with our results, former reports showed that germination and fermentation processes augmented the protein contents of various seeds [35,39,41,45,48]. The reduction in moisture content in the fermented samh seeds is probably due to the increase in total solids, which is linked to an increase in the mass of fermenting microorganisms. The moisture content of the raw and fermented samh seeds was ≤10 and thus considered safe for storage [45], whereas that of the germinated samh seeds was higher than the safe level, and increased moisture in the germinated seeds could be due to increased water uptake during germination. The reduced fiber level in the germinated and fermented samh seeds is likely due to the enzymatic breakdown of fiber during fermentation and germination processes, and a similar reduction in fiber from fermented and germinated seeds has been reported [45,48]. The increase in fat content in the fermented seeds could be due to the breakdown of complex constituents which led to the release of more lipids and the synthesis of lipids by yeast and molds during the fermentation process, whereas the reduced fat in the germinated seeds is likely due to the enzymatic hydrolysis of lipids into fatty acids and glycerol [48]. The reduction in carbohydrates in the germinated and fermented samh seeds might be accredited to metabolic hydrolysis and the consumption of carbohydrates as an energy source by microorganisms, and a similar reduction in carbohydrates during fermentation and germination has been reported in various studies [48,49,50]. It is worth noting that the estimated glycemic indices of the raw, germinated, and fermented samh seeds were 41.43, 40.07, and 39.61, indicating that it is a low glycemic food based on the International Standards Organization (ISO) classification of foods as high (GI ≥ 70), medium (GI = 56–69), and low (GI ≤ 55) glycemic foods [51]. In nutrition, high, medium, and low glycemic index foods are categorized as good, better, and best choices [52], and based on that classification, samh seeds could be considered the best choice for diabetic people. During fermentation and germination treatments, the glycemic index of the samh seeds was reduced, suggesting a more beneficial effect of these treatments on the nutritional quality of samh seeds. The reduction in the estimated glycemic index of the germinated and fermented samh seeds is probably due to the regulation of glucose metabolism by bioactive compounds released during fermentation and germination processes, namely peptides, phenolic compounds, and amino acids, in addition to increased acidity, which reduced starch hydrolysis [53]. In accordance with our findings, previous reports demonstrated that germination and fermentation treatments reduced the estimated glycemic index of chickpea and purple potato flour [50,53].

### 3.3. The Effect of Fermentation and Germination on the Bioactive Properties and Antinutritional Factors of Samh Seeds

Table 3 shows the results of phenolic compounds, total flavonoid content (TFC), total phenolic content (TPC), antioxidant activity (DPPH, FRAP, and ABTS), and antinutritional factors (phytate and tannin content) of the raw, germinated, and fermented samh seeds. Generally, both treatments significantly (*p* < 0.05) enhanced the phenolic compounds, TFC, and TPC of the samh seeds, and consequently, the antioxidant activity as assessed by the ABTS, DPPH, and FRAP approaches was also greatly (*p* < 0.05) improved by both treatments. The highest TFC was observed in the germinated seeds followed by the fermented seeds, whereas the lowest values were observed in the raw seeds (*p* < 0.05). The highest levels of TPC, ABTS, DPPH, and FRAP were found in the fermented seeds followed by the germinated seeds, whereas the lowest values were observed in the raw samples (*p* < 0.05). Among the phenolic compounds (Table 3 and Figure S1), gallic acid was the most abundant compound in the samh seeds samples followed by catechol, catechin, and protocatechuic acid, and the content of all phenolic compounds was augmented by the fermentation and germination processes. Generally, phenolic acids were high in the fermented samh seeds, whereas flavonoids were high in the germinated samh seeds. During the germination and fermentation processes, different metabolic mechanisms occur, leading to various modifications of the bioactive properties of germinated and fermented products. Fermenting microbes could produce several cell wall-degrading enzymes such as pectinases, xylanase, β-glucosidase, and esterase and thereby liberate more free forms of phenolic compounds in fermented products [40]. In addition, during germination several signaling pathways such as shikimate, tannin hydrolysis, glycolysis, and pentose phosphate pathways are developed and activated for the de novo creation of phenolic compounds [54]. Therefore, the increase in TPC, TFC, and phenolic compounds of the germinated and fermented samh seeds is likely due to the activity of fermenting microbes and enzymes leading to the release of bound phenolic compounds and/or the synthesis and generation of new phenolic compounds. The improved antioxidant activity (DPPH, ABTS, and FRAP) of the germinated and fermented samh seeds is likely due to the upsurge in phenolic compounds during the fermentation and germination processes. In agreement with our findings, many investigators have reported similar changes in the bioactive properties of various seeds and grains following germination and fermentation processes [40,43,45,55].

Fermentation and germination treatments led to a substantial (*p* < 0.05) reduction in the tannin and phytate contents compared to the control. The uppermost levels of tannin and phytate were witnessed in the raw samh seeds followed by the fermented and then germinated seeds. The highest decline in phytate and tannin was evident in the germinated seeds. The decrease in phytate and tannin content in the fermented and germinated samh seeds could be ascribed to the augmented metabolic activity of tannin- and phytate-degrading enzymes such as tannin acyl hydrolases, phosphatases, phytases, and polyphenol oxidases during the germination and/or fermentation of samh seeds [40]. Previously, several investigators reported that germination and fermentation resulted in a reduction of tannins and phytate in grains and seeds [40,56]. Tannins and phytate are known to have a negative impact on the availability of nutrients such as minerals, starch, and proteins due to their chelating ability and inhibiting effects on metabolic enzymes. Therefore, a reduction in such antinutrients in samh seeds by fermentation and germination could improve the nutritional quality in addition to the positive influences of these processes on the bioactive properties of samh seeds, which could improve the health potentials of the products.

### 3.4. The Effect of Germination and Fermentation on the Amino Acids Profile and Protein Digestibility of Samh Seeds

The effect of germination and fermentation on the amino acids profile and in vitro protein digestibility of the samh seeds is illustrated in Table 4 and Figure S2. It was noted that germination and fermentation affected the essential and non-essential amino acids of samh seeds differently. With the exception of threonine, germination treatment considerably (*p* < 0.05) augmented the essential and non-essential amino acids of samh seeds to the highest levels compared to the raw and fermented samples, which might be associated with a high reduction in antinutritional factors in germinated seeds (Table 3). The upsurge in amino acids in germinated seeds is likely due to modification of the globular structure during the germination process, resulting in increased enzyme accessibility, hydrolysis of proteins and polypeptides, and liberation of bound amino acids [57,58]. Similarly, a previous study showed that germination augmented the essential and non-essential amino acids of Bambara groundnut, with the exception of arginine, which was reduced during the germination process [45]. Additionally, the germination treatment augmented all essential and non-essential amino acids of pigeon pea, which was ascribed to a reduction in antinutritional factors, the enzymatic degradation of proteins and polypeptides, and the liberation of bound amino acids during the germination process [59]. Moreover, germination increased the free amino acids of different quinoa varieties [60] and the essential amino acids of Tartary buckwheat, except for valine [56], and these changes were attributed to the enzymatic hydrolysis of protein and starch and the release and synthesis of amino acids to meet the metabolic needs of the germinated seeds. With the exception of histidine and lysine, the fermentation process augmented (*p* < 0.05) all essential amino acids of the samh seeds compared to the control, which might be ascribed to the production of essential amino acids by fermenting microbes, especially lactic acid bacteria [35]. However, it reduced the amounts of non-essential amino acids in the samh seeds, except glutamic acid and arginine, which were greater in the fermented samples than in the control ones (*p* < 0.05). The reduction in non-essential amino acids during the fermentation of the samh seeds could be due to the metabolisms of amino acids by fermenting microbes [61]. Similarly, it has been stated that LAB fermentation increased the essential amino acids of sorghum flour [62]. In contrast, fermentation augmented the amounts of both essential and non-essential amino acids in sorghum flour [35] and African yam bean flour [44]. The total essential amino acids of samh seeds also increased from 38.16 in raw seeds to 41.24 and 46.0 in fermented and germinated seeds, respectively. The percent increase in total essential amino acids was 8.07% in the fermented seeds and 20.55% in the germinated seeds. The total non-essential amino acids also increased significantly from 49.80% in the raw seeds to 52.15% and 71.73% (4.72% and 44.04% percent increase) in the fermented and germinated seeds, respectively. The total amino acid content was also augmented from 87.96% in the raw samh seeds to 93.39% in the fermented seeds and 117.73% in the germinated seeds. The ratio of essential amino acids to the total amino acids (EAA/TAA) was high in the fermented seeds (44.16%) followed by the raw seeds (43.38), whereas it was low in the germinated seeds (39.07%). This signifies the improvement in essential amino acids of samh seeds during the fermentation process and the loss of essential amino acids during the germination process. It is worth noting that the EAA/TAA of all samples was >39%, which was considered adequate as stated by the WHO in that the acceptable levels of EAA/TAA for infants, children, and adults were above 39%, 26%, and 12%, respectively [35]. These findings indicated that the EAA/TAA of the samh seeds with and without fermentation and germination was adequate for all groups of people.

The in vitro digestibility of samh seeds protein was considerably (*p* < 0.05) enhanced by germination and fermentation treatments, with the highest level of protein digestibility being observed in the fermented seeds (57.28%) followed by the germinated seeds (51.04%), whereas the lowest value was seen in the raw seeds (45.63%). The changes in the in vitro protein digestibility of the samh seeds were shown in a 25.53% and 11.86% increase in the fermented and germinated seeds, respectively. The increase in the protein digestibility of the samh seeds following germination and fermentation treatments is probably due to the modification of native proteins, alteration of the interaction between proteins and antinutritional factors, and the availability of protein functional groups that are susceptible to enzymatic hydrolysis [45,63]. Similarly, previous reports established that fermentation and germination processes enhanced the in vitro protein digestibility of various cereal and legume grains [45,59,63]. The predicted protein efficiency ratio (P-PER) of the samh seeds also increased (*p* < 0.05) from 0.36 in the raw seeds to 0.49 and 1.06 in the fermented and germinated seeds, respectively, indicating better biologically available proteins in the germinated samh seeds followed by the fermented samh seeds. Similarly, an improved protein efficiency ratio was reported in germinated sorghum grains [35] and fermented quinoa flour [64] compared to untreated samples. Overall, fermentation and germination enhance the amino acids profile and the in vitro protein digestibility of the samh seeds and could thus pave the way for the utilization of fermented and germinated samh seeds for developing nutritious foods.

### 3.5. The Effect of Fermentation and Germination on the Minerals Content of Samh Seeds

The effect of fermentation and germination on the mineral content of raw, germinated, and fermented samh seeds is shown in Table 5. Among the macro-minerals, fermentation treatment significantly (*p* < 0.05) augmented the levels of calcium, magnesium, sodium, and phosphorous to the highest levels compared to the control. The uppermost level of potassium was apparent in the germinated seeds followed by the fermented seeds. Germination reduced the levels of calcium, magnesium, and sodium and increased the phosphorous content of the samh seeds compared to the control (*p* < 0.05). Among the micro-minerals, the uppermost levels of chromium, iron, and zinc were seen in the fermented seeds, whereas the uppermost amounts of boron, copper, and manganese were seen in the raw seeds. Fermentation treatment increased the levels of the assessed micro-minerals, except boron and copper, which were reduced by fermentation compared to those of the raw seeds. Germination treatment reduced the levels of the assessed micro-minerals, except zinc and chromium, which increased after germination compared to the untreated control (*p* < 0.05). Generally, fermentation increased the mineral content of the samh seeds, with a few exceptions, whereas germination reduced most of the assessed minerals, except potassium, chromium, and zinc, which were higher in the germinated seeds compared to the raw ones. The increase in minerals following the fermentation treatment of the samh seeds is probably due to the degradation of antinutritional factors and the liberation of bound minerals into soluble forms during the fermentation process [41,48,59]. However, the reduction in minerals during germination treatment is likely due to the utilization of these minerals as cofactors for some enzymes that are involved in the catalysis of macromolecules, in addition to the leaching out of some minerals during the steeping of the samh seeds [56]. Previous reports indicated that fermentation and germination exhibited positive and negative influences on the mineral contents of several seeds and grains [41,44,45,48,59,65].

### 3.6. The Effect of Fermentation and Germination on the Fatty Acids of Samh Seeds

The results of the fatty acid composition of samh seeds as influenced by fermentation and germination treatments are presented in Table 6. Fermentation and germination treatments impacted the fatty acids of samh seeds in different manners (*p* < 0.05). The most dominant fatty acids in the raw, fermented, and germinated samh seeds were linolenic acid followed by oleic and palmitic acids, which is in agreement with the results of Al-Jassir et al. [7], who stated that linolenic acid followed by oleic and palmitic acids are the most abundant fatty acids in samh seeds. In addition, Mohamed Ahmed et al. [5] stated that linolenic acid was the most dominant fatty acid followed by elaidic and palmitic acids in raw and roasted samh seeds, which is somewhat comparable to the results of the current study. Variations in the environmental conditions, growing season, growing area, and soil type and fertility could be the reasons for slight differences in fatty acid composition between these studies [5]. Among the saturated fatty acids, the uppermost levels of palmitic acid and stearic acids were found in the fermented and germinated samples, respectively, whereas the other acids were not different among the raw, germinated, and fermented samples. The highest amounts of total saturated fatty acids were seen in the fermented samh seeds, whereas the lowest value was seen in the germinated sample. Myritic acid and behenic acid were not detected in the germinated samples. Germination significantly reduced palmitic acid and total saturated fatty acids and increased stearic acids compared to the unfermented samh seeds. Fermentation caused a significant increase in palmitic acid and total saturated fatty acids and a slight upsurge in stearic and behenic acids, whereas it showed a slight reduction in arachidic acid. Among the unsaturated fatty acids, the uppermost levels of oleic acid and total unsaturated fatty acids were observed in the germinated samh seeds, whereas the lowest values were seen in the fermented samh seeds. The highest levels of linoleic acid were observed in the raw and fermented samh seeds and those of linolenic acid were seen in the raw and germinated samh seeds. Linoelaidic acid was not detected in the germinated seeds. It should be noted that the germination process increased the total unsaturated fatty acids to the highest value, whereas fermentation increased the total saturated fatty acids to the highest value. Generally, unsaturated fatty acids were more abundant than saturated fatty acids in the samh seeds, with them having a ratio of 5.05, 4.51, and 4.77 in the germinated, fermented, and raw samh seeds, respectively, which is similar to previous findings [5]. The changes in fatty acid contents in the fermented and germinated samh seeds is likely due to the metabolic activity of lipase and phospholipase enzymes during germination and fermentation processes leading to an increase and/or a decrease in the individual fatty acids of samh seeds [63,66]. During germination and fermentation processes, lipids are utilized for energy through the hydrolysis of triacylglycerols, diacylglycerols, and monoacylglycerols, thereby increasing the release and accumulation of individual fatty acids in fermented and germinated seeds. Further utilization of fatty acids for energy generation during fermentation and germination processes led to a reduction in fatty acids in germinated and fermented seeds [67,68]. Variable changes (increase, decrease, or no change) in fatty acid composition have been reported during the germination and fermentation of various grains and seeds, and the variations are ascribed to the alterations in the germination and fermentation conditions and grains and seed types [63,67,68]. Overall, the outcomes of this study demonstrate that samh seeds are rich in unsaturated fatty acids (>81.5%), revealing the high nutritional quality of samh seeds, which was greatly enhanced by the germination process and slightly reduced by the fermentation process. In addition, the great amounts of linoleic acid and polyunsaturated fatty acids in the raw, fermented, and germinated samh seeds prove the nutritional and health potentials of these seeds in the prevention of cancer and cardiovascular diseases [68].

## 4. Conclusions

In this study, the impacts of fermentation and germination on the functional, nutritional, and health quality attributes of samh seeds were evaluated. Samh seeds are rich in bioactive compounds, protein, essential amino acids, and unsaturated fatty acids and could be deliberated as a promising candidate for developing functional foods. Germination and fermentation treatments can enhance the nutritional value of samh seeds by increasing their bioactive properties (TPC, TFC, phenolic compounds, and antioxidant activity), in vitro protein digestibility, protein efficiency ratio, and total essential amino acids and reducing antinutritional factors (tannin and phytate), carbohydrate content, and glycemic index. In addition, both processes could also improve the functional characteristics of samh seeds by their increasing oil absorption capacity (OAC), water absorption capacity (WAC), foaming capacity, and swelling power. Germination also enhanced the nutritional quality of the samh seeds by increasing potassium, zinc, phosphorous, and oleic and unsaturated fatty acids and reducing fat content, palmitic acid, and total saturated fatty acids. Fermentation also enhanced the nutritional quality of samh seeds by increasing protein, calcium, magnesium, sodium, phosphorous, iron, zinc, palmitic acid, and total saturated fatty acids. However, it reduced the moisture, non-essential amino acids, and total unsaturated fatty acids of the samh seeds. Overall, samh seeds are a rich source of nutrients and bioactive compounds, which are mostly enhanced by germination and fermentation processes. Fermented and/or germinated samh seeds can be used in food applications as nutritious and functional food, adding more value to these underutilized wild plant seeds.

## Figures and Tables

**Table 1:** Effect of fermentation and germination on the color and functional properties of samh seeds.

	Color			Functional Properties		
Sample	b*	a*	L*	WAC (mL/100 g)	OAC (mL/100 g)	Emulsion Activity (%)
Raw	9.82 ± 0.02 ^c^	8.36 ± 0.05 ^b^	30.90 ± 0.00 ^c^	29.01 ± 0.21 ^c^	24.45 ± 0.15 ^c^	38.31 ± 0.21 ^b^
Fermented	13.34 ± 0.02 ^a^	8.26 ± 0.03 ^c^	33.70 ± 0.01 ^a^	32.18 ± 0.36 ^a^	26.83 ± 0.24 ^a^	43.09 ± 0.32 ^a^
Germinated	13.03 ± 0.23 ^b^	8.61 ± 0.04 ^a^	32.97 ± 0.29 ^b^	30.93 ± 0.74 ^b^	25.23 ± 0.87 ^b^	36.23 ± 0.26 ^c^
% Change F	35.8	−1.2	9.1	10.9	9.7	12.5
% Change G	32.69	2.99	6.70	6.62	3.19	−5.43
	**Functional properties**					
	**Emulsion stability (%)**	**Foaming capacity (%)**	**Foaming stability (%)**	**Bulk density (g/cm^3^)**	**Swelling power (g/g)**	**Water solubility (%)**
Raw	37.12 ± 0.28 ^a^	15.93 ± 0.25 ^c^	38.31 ± 0.98 ^b^	0.795 ± 0.52 ^b^	2.33 ± 0.17 ^c^	3.35 ± 0.11 ^a^
Fermented	34.06 ± 0.36 ^c^	24.06 ± 0.78 ^a^	43.09 ± 0.12 ^a^	0.813 ± 0.38 ^a^	2.64 ± 0.19 ^a^	2.70 ± 0.42 ^b^
Germinated	35.09 ± 0.13 ^b^	17.46 ± 0.95 ^b^	36.23 ± 0.41 ^c^	0.798 ± 0.18 ^b^	2.49 ± 0.12 ^b^	1.98 ± 0.21 ^c^
% Change F	−8.2	51.0	12.5	2.3	13.3	−19.4
% Change G	−5.47	9.60	−5.43	0.38	6.87	−40.90

The values are the means of three replications ± standard deviation. Differences in the superscript letter(s) in each column indicate statistical variations at *p* < 0.05.

**Table 2:** Effect of fermentation and germination on the pH, acidity, total solids, total viable count (TVC), yeast and mold counts, proximate composition, fiber, and glycemic index of samh seeds.

Sample	Total Solids (%)	Acidity (g Lactic Acid/100 g)	pH	TVC (Log CFU/g)	Yeast and Mold (Log CFU/g)	Ash (%)
Raw	91.47 ± 0.25 ^b^	0.30 ± 0.05 ^b^	6.12 ± 0.01 ^a^	6.08 ± 0.03 ^c^	2.27 ± 0.02 ^c^	3.53 ± 0.29 ^a^
Fermented	94.41 ± 0.26 ^a^	2.27 ± 0.10 ^a^	4.43 ± 0.03 ^c^	7.60 ± 0.04 ^a^	3.91 ± 0.11 ^a^	3.33 ± 0.01 ^ab^
Germinated	88.73 ± 0.21 ^c^	0.45 ± 0.09 ^b^	6.08 ± 0.02 ^b^	6.40 ± 0.02 ^b^	3.61 ± 0.12 ^b^	3.11 ± 0.04 ^b^
% Change F	3.2	656.7	−27.6	25.0	72.2	−5.7
% Change G	−3.00	50.00	−0.65	5.26	59.03	−11.90
	**Mositure (%)**	**Fat (%)**	**Protein (%)**	**Carbohydrate (%)**	**Fiber (%)**	**Estimated glycemic index (eGI)**
Raw	8.52 ± 0.26 ^b^	5.47 ± 0.31 ^b^	19.29 ± 0.27 ^b^	63.19 ± 0.16 ^a^	4.36 ± 0.07 ^a^	41.43 ± 0.44 ^a^
Fermented	7.58 ± 0.01 ^c^	6.51 ± 0.11 ^a^	20.41 ± 0.49 ^a^	62.16 ± 0.04 ^b^	2.45 ± 0.05 ^c^	39.61 ± 0.29 ^c^
Germinated	11.26 ± 0.21 ^a^	4.88 ± 0.08 ^c^	19.67 ± 0.48 ^ab^	61.07 ± 0.12 ^c^	3.38 ± 0.08 ^b^	40.07 ± 0.05 ^b^
% Change F	−11.0	19.0	5.8	−1.6	−43.8	−4.4
% Change G	32.16	−10.79	1.97	−3.35	−22.48	−3.28

The values are the means of three replications ± standard deviation. Differences in superscript letter(s) in each column indicate statistical variations at *p* < 0.05.

**Table 3:** Effect of fermentation and germination on the bioactive properties, phenolic compounds, and antinutritional factors of samh seeds.

Properties	Raw	Fermented	Germinated	% Change F	% Change G
Bioactive properties					
TPC (mg GAE/g DM)	1.21 ± 0.04 ^c^	2.43 ± 0.06 ^a^	1.72 ± 0.07 ^b^	100.8	42.15
TFC (mg Catechin/g DM)	1.60 ± 0.33 ^c^	2.56 ± 0.34 ^b^	3.69 ± 0.17 ^a^	60.0	130.63
FRAP (mg Trolox/g DM)	0.77 ± 0.01 ^c^	0.90 ± 0.02 ^a^	0.85 ± 0.01 ^b^	16.9	10.39
ABTS (mg Trolox/g DM)	0.66 ± 0.05 ^b^	0.81 ± 0.03 ^a^	0.75 ± 0.09 ^ab^	22.7	13.64
DPPH inhibition (%)	0.65 ± 27.61 ^c^	78.39 ± 0.57 ^a^	74.03 ± 0.32 ^b^	20.1	13.42
Phenolic compounds (mg/100 g)					
Gallic acid	79.6 ± 0.11 ^c^	85.02 ± 0.42 ^b^	96.36 ± 1.75 ^a^	6.81	21.06
Protocatechuic acid	32.21 ± 1.15 ^b^	44.48 ± 0.55 ^a^	43.64 ± 1.25 ^a^	38.09	35.49
Caffeic acid	3.37 ± 0.10 ^c^	4.72 ± 0.07 ^a^	3.97 ± 0.09 ^b^	40.06	17.80
Syringic acid	5.16 ± 0.30 ^c^	8.28 ± 0.06 ^a^	6.93 ± 0.09 ^b^	60.47	34.30
p-Coumaric acid	0.44 ± 0.02 ^c^	0.78 ± 0.05 ^a^	0.62 ± 0.03 ^b^	77.27	40.91
trans-Ferulic acid	2.33 ± 0.12 ^c^	5.26 ± 0.05 ^a^	4.54 ± 0.23 ^b^	125.75	94.85
trans-Cinnamic acid	0.43 ± 0.06 ^c^	0.69 ± 0.02 ^b^	1.02 ± 0.06 ^a^	39.53	137.21
Catechol	56.34 ± 1.21 ^c^	70.34 ± 1.06 ^b^	77.34 ± 0.78 ^a^	24.85	37.27
Catechin	49.15 ± 2.13 ^c^	63.67 ± 1.17 ^b^	84.93 ± 1.03 ^a^	29.54	72.80
Rutin trihydrate	0.30 ± 0.05 ^c^	1.19 ± 0.09 ^b^	1.04 ± 0.32 ^b^	296.67	246.67
Apigenin 7 glucoside	0.45 ± 0.01 ^c^	1.00 ± 0.08 ^b^	1.87 ± 0.06 ^a^	122.22	315.56
Quercetin	1.51 ± 0.10 ^c^	4.52 ± 0.13 ^b^	5.40 ± 0.07 ^a^	199.34	257.62
Resveratrol	0.32 ± 0.02 ^b^	0.58 ± 0.08 ^a^	0.65 ± 0.05 ^a^	81.25	103.13
Kaempferol	4.13 ± 0.05 ^c^	6.51 ± 0.03 ^b^	7.56 ± 0.01 ^a^	57.63	83.05
Isorhamnetin	2.13 ± 0.66 ^c^	4.51 ± 0.21 ^b^	5.56 ± 0.04 ^a^	111.74	161.03
Antinutritional factors (ANFs)					
Tannins (mg TAE/g DM)	0.83 ± 0.08 ^a^	0.45 ± 0.06 ^b^	0.26 ± 0.08 ^c^	−45.8	−68.67
Phytic acid (mg/g DM)	0.88 ± 0.07 ^a^	0.35 ± 0.06 ^b^	0.22 ± 0.08 ^b^	−60.2	−75.00

The values are the means of three replications ± standard deviation. Differences in superscript letter(s) in each row indicate statistical variations at *p* < 0.05.

**Table 4:** Effect of fermentation and germination on the amino acid composition and protein digestibility of samh seeds.

Amino Acid (g/100 g DM)	Raw	Fermented	Germinated	% Change F	% Change G
Essential amino acids					
Histidine	2.73 ± 0.15 ^b^	2.66 ± 0.15 ^c^	3.44 ± 0.21 ^a^	−2.56	26.01
Threonine	15.84 ± 0.21 ^b^	17.22 ± 0.21 ^a^	15.21 ± 0.15 ^c^	8.71	−3.98
Valine	8.53 ± 0.15 ^c^	8.82 ± 0.13 ^b^	9.86 ± 0.19 ^a^	3.40	15.59
Methionine	2.54 ± 0.06 ^c^	2.97 ± 0.32 ^b^	4.73 ± 0.09 ^a^	16.93	86.22
Phenylalanine	3.77 ± 0.20 ^c^	4.36 ± 0.14 ^b^	5.44 ± 0.14 ^a^	15.65	44.30
Isoleucine	1.25 ±0.15 ^b^	1.76 ± 0.15 ^a^	1.78 ± 0.15 ^a^	40.80	42.40
Leucine	2.35 ± 0.11 ^c^	2.51 ± 0.12 ^b^	4.06 ± 0.11 ^a^	6.81	72.77
Lysine	1.15 ± 0.15 ^b^	1.12 ± 0.11 ^b^	1.48 ± 0.16 ^a^	−2.61	28.70
Total essential amino acids (TEAA)	38.16 ±1.10 ^c^	41.24 ± 1.37 ^b^	46.00 ± 1.19 ^a^	8.07	20.55
Nonessential amino acids					
Aspartic acid	4.03 ± 0.20 ^b^	3.63 ± 0.25 ^c^	4.42 ± 0.15 ^a^	−9.93	9.68
Glutamic acid	8.14 ± 0.15 ^c^	8.46 ± 0.15 ^b^	10.64 ± 0.23 ^a^	3.93	30.71
Serine	2.12 ± 0.15 ^b^	1.37 ± 0.20 ^c^	2.82 ± 0.16 ^a^	−35.38	33.02
Glycine	2.17 ± 0.13 ^b^	1.37 ± 0.17 ^c^	2.81 ± 0.11 ^a^	−36.87	29.49
Arginine	5.18 ± 0.35 ^c^	6.06 ± 0.05 ^b^	10.24 ± 0.32 ^a^	16.99	97.68
Alanine	1.73 ± 0.20 ^b^	1.22 ± 0.13 ^c^	2.03 ± 0.17 ^a^	−29.48	17.34
Tyrosine	2.32 ± 0.22 ^b^	1.73 ± 0.21 ^c^	3.03 ± 0.25 ^a^	−25.43	30.60
Proline	24.13 ± 0.20 ^b^	28.31 ± 0.10 ^c^	35.74 ± 0.21 ^a^	17.32	48.11
Total non-essential amino acids (TNEAA)	49.80 ± 1.10 ^c^	52.15 ± 1.06 ^b^	71.73 ± 1.89 ^a^	4.72	44.04
Total amino acids (TAA)	87.96 ± 1.34 ^c^	93.39 ± 2.20 ^b^	117.73 ± 1.67 ^a^	6.17	33.84
EAA/TAA (%)	43.38	44.16	39.07	1.80	−9.94
Predicted protein efficiency ratio (P-PER)	0.36	0.49	1.06	36.11	194.44
In vitro protein digestibility IVPD (%)	45.63 ± 0.72 ^c^	57.28 ± 0.67 ^a^	51.04 ± 0.62 ^b^	25.53	11.86

The values are the means of three replications ± standard deviation. Differences in superscript letter(s) in each row indicate statistical variations at *p* < 0.05. EAA/TAA, essential-to-total amino acids ratio.

**Table 5:** Effect of fermentation and germination on the mineral composition of the samh seeds.

Minerals (mg/kg DM)	Raw	Fermented	Germinated	% Change F	% Change G
Macro-minerals					
Ca	13.6 ± 1.2 ^b^	15.7 ± 0.8 ^a^	12.8 ± 0.4 ^c^	15.44	−5.88
Mg	55.06 ± 1.1 ^b^	61.96 ± 2.4 ^a^	45.20 ± 2.1 ^c^	12.53	−17.91
K	47.1 ± 1.2 ^c^	56.1 ± 3.3 ^b^	63.8 ± 0.9 ^a^	19.11	35.46
Na	12.7 ± 0.8 ^a^	13.3 ± 0.3 ^a^	10.0 ± 0.6 ^b^	4.72	−21.26
P	71.03 ± 0.4 ^b^	77.58 ± 0.9 ^a^	71.88 ± 0.7 ^b^	9.22	1.20
Micro-minerals					
B	0.22 ± 0.05 ^a^	0.12 ± 0.02 ^b^	0.05 ± 0.04 ^c^	−45.45	−77.27
Cr	0.09 ± 0.01 ^c^	0.24 ± 0.03 ^a^	0.15 ± 0.03 ^b^	166.67	66.67
Cu	0.15 ± 0.03 ^a^	0.12 ± 0.1 ^b^	0.07 ± 0.01 ^c^	−20.00	−53.33
Fe	2.75 ± 0.1 ^b^	3.48 ± 0.1 ^a^	2.32 ± 0.1 ^c^	26.55	−15.64
Mn	2.64 ± 0.1 ^a^	2.63 ± 0.2 ^a^	2.08 ± 0.1 ^b^	−0.38	−21.21
Zn	0.37 ± 0.2 ^c^	0.47 ± 0.01 ^a^	0.42 ± 0.02 ^b^	27.03	13.51

The values are the means of three replications ± standard deviation. Differences in superscript letter(s) in each row indicate statistical variations at *p* < 0.05.

**Table 6:** Effect of fermentation and germination on the fatty acids composition of samh seeds.

Fatty Acids (% of Oil)	Raw	Fermented	Germinated	% Change F	% Change G
Saturated fatty acids (SFA)					
Myristic (C14:0)	0.25 ± 0.01 ^a^	0.23 ± 0.01 ^a^	ND	−8.00	−100.00
Palmitic (C16:0)	13.40 ± 0.05 ^b^	14.14 ± 0.30 ^a^	12.95 ± 0.03 ^c^	5.52	−3.36
Stearic (C18:0)	2.63 ± 0.02 ^b^	2.65 ± 0.06 ^b^	2.94 ± 0.04 ^a^	0.76	11.79
Arachidic (C20:0)	0.72 ± 0.04 ^a^	0.70 ± 0.04 ^a^	0.65 ± 0.03 ^a^	−2.78	−9.72
Behenic (C22:0)	0.33 ± 0.03 ^a^	0.34 ± 0.04 ^a^	ND	3.03	−100.00
ΣSFA	17.33 ± 0.19 ^b^	18.16 ± 0.33 ^a^	16.54 ± 0.45 ^c^	4.79	−4.56
Unsaturated fatty acids (USFA)					
Oleic (C18:1 cis- ω 9)	26.99 ± 0.17 ^b^	26.52 ± 0.18 ^c^	28.81 ± 0.03 ^a^	−1.74	6.74
Linolelaidic (C18:2 trans, trans- ω 6)	0.25 ± 0.03 ^a^	0.29 ± 0.02 ^a^	ND	16.00	−100.00
Linoleic acid (C18:2 cis, cis- ω 6)	54.24 ± 0.27 ^a^	54.06 ± 0.06 ^a^	53.47 ± 0.12 ^b^	−0.33	−1.42
Linolenic (C18:3- ω 3)	1.19 ± 0.01 ^a^	1.08 ± 0.01 ^b^	1.18 ± 0.04 ^a^	−9.24	−0.84
ΣUSFA	82.67 ± 0.12 ^b^	81.95 ± 0.33 ^c^	83.46 ± 0.24 ^a^	−0.87	0.96
ΣPUFA	55.68	55.43	54.65	−0.45	−1.85
USFA/SFA	4.77	4.51	5.05	−5.45	5.87
PUFA/SFA	3.21	3.05	3.30	−4.98	2.80
ω 3	1.19	1.08	1.18	−9.24	−0.84
ω 6	54.49	54.35	53.47	−0.26	−1.87
ω 9	26.99	26.52	28.81	−1.74	6.74
ω 6/ω 3	45.79	50.32	45.31	8.89	−1.05

The values are the means of three replications ± standard deviation. Differences in superscript letter(s) in each row indicate statistical variations at *p* < 0.05. ND: not detected.

## Data Availability

Data are contained within the article.

## Supplementary Materials

The following supporting information can be downloaded at: https://www.mdpi.com/article/10.3390/foods12224133/s1, Figure S1: Phenolic compounds of raw, fermented and germinated samh seeds; Figure S2: Amino acid profile of raw, fermented, and germinated samh seeds.
