# Supplementary material for: The Effect of Germination and Fermentation on the Physicochemical, Nutritional, and Functional Quality Attributes of Samh Seeds
Source: Foods. 2023 Nov 15;12(22):4133. doi: 10.3390/foods12224133 (PMC10669962; doi:10.3390/foods12224133)
Supplement: Supplementary file 1 [file foods-12-04133-s001.zip › foods-2684722-supplementary.pdf]

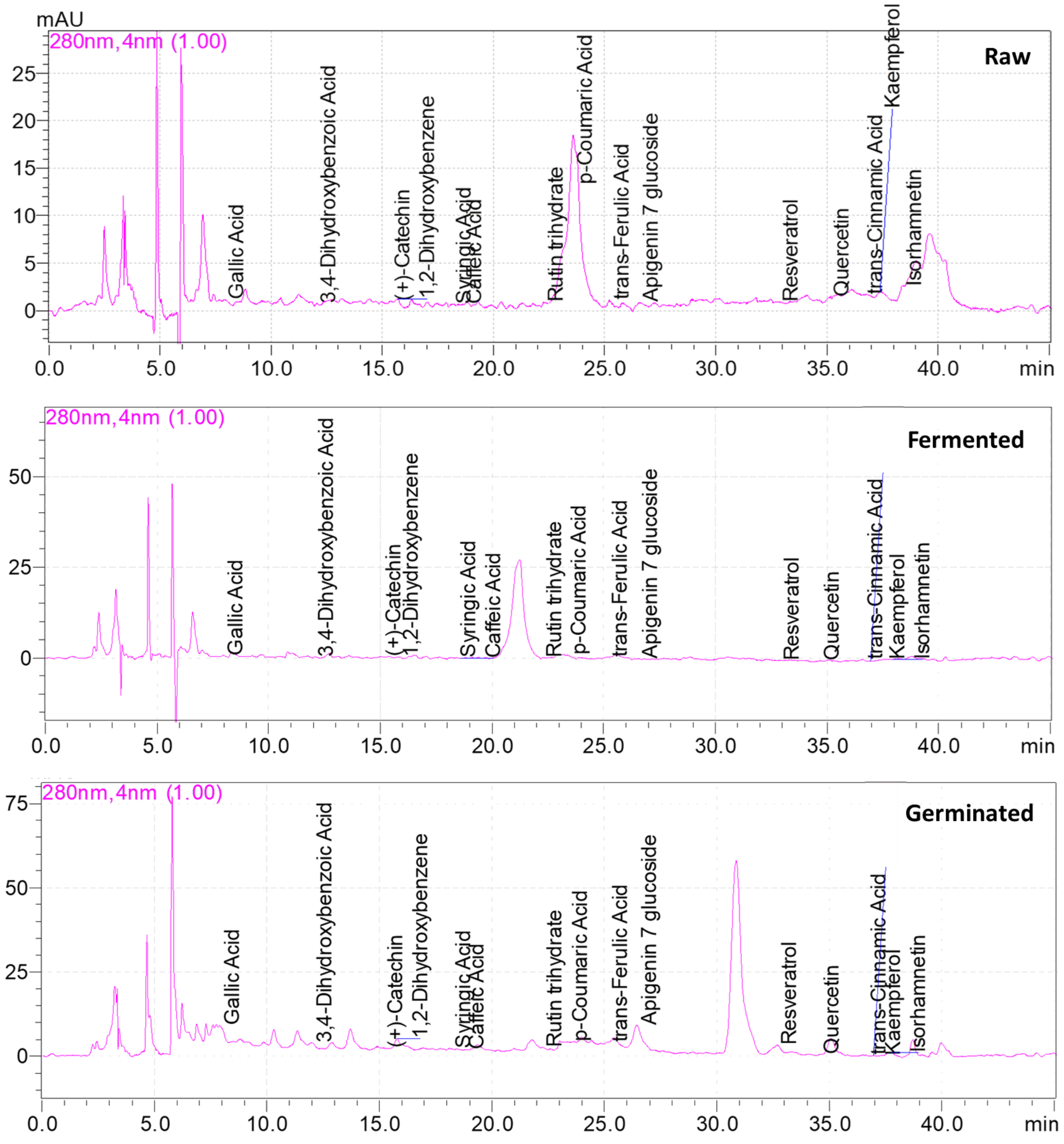

Figure S1. Phenolic compounds of raw, fermented and germinated samh seeds.

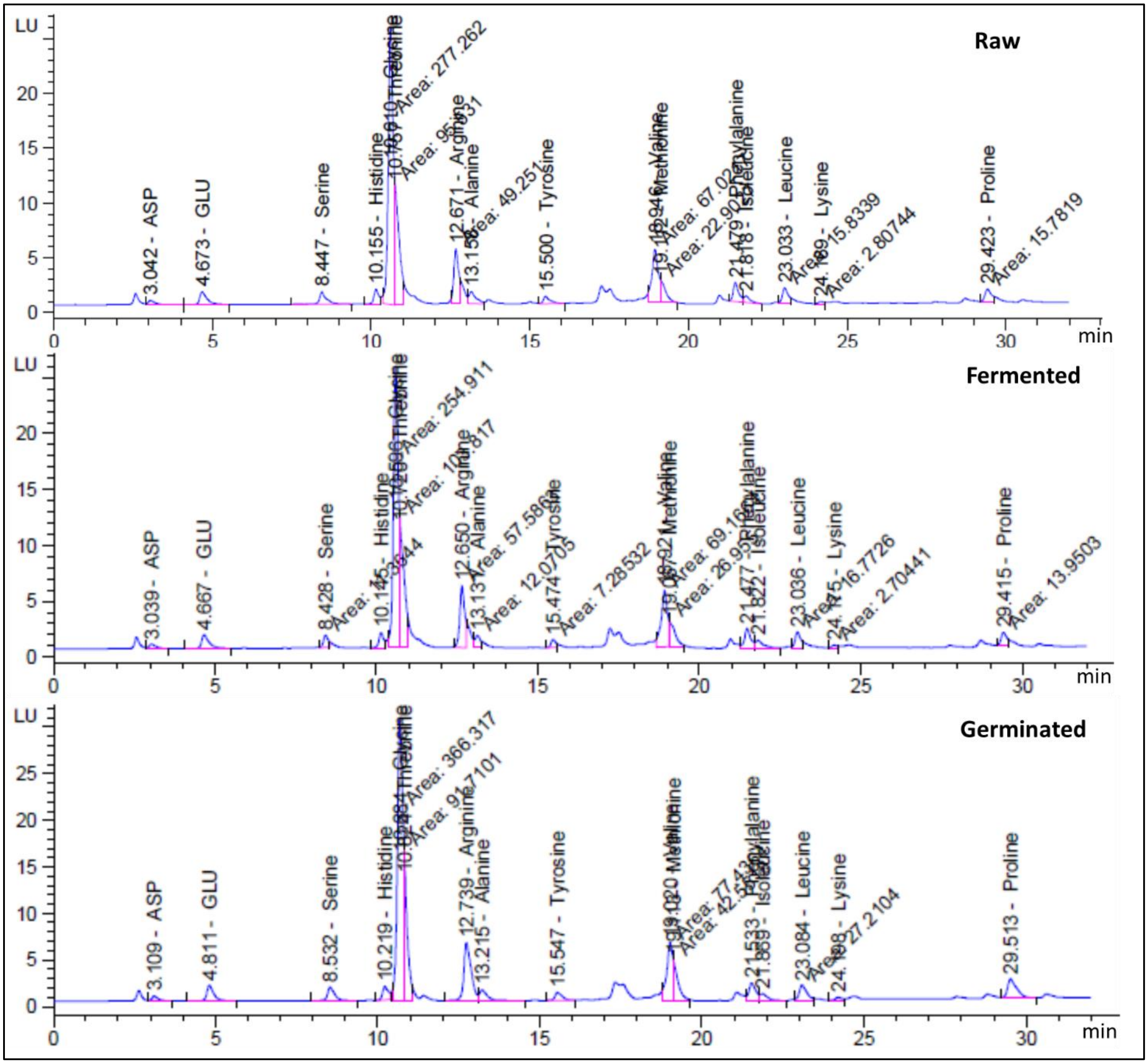

Figure S2. Amino acid profile of raw, fermented, and germinated samh seeds
